# Finger Enthesophytes at the Flexor Digitorum Superficialis Insertion in an Avid Bowler: A Case Report

**DOI:** 10.7759/cureus.113075

**Published:** 2026-07-21

**Authors:** Ian K Christman, Alexandre G Vesselle, Julian Gatta

**Affiliations:** 1 Orthopaedic Surgery, University Hospitals Cleveland Medical Center, Cleveland, USA

**Keywords:** bowling, enthesis, enthesitis, enthesophytes, flexor digitorum superficialis

## Abstract

Enthesophytes are bony spurs that form at the insertion sites of ligaments, tendons, or articular capsules on bone, also known as entheses. In the hand, they commonly form due to inflammatory disorders but can also form due to mechanical stress. It is hypothesised that the mechanical loads cause micro-damage of the fibrocartilaginous enthesis, which results in new bone formation from osteoblast differentiation. We present a unique case where a patient formed enthesophytes at the flexor digitorum superficialis (FDS) insertion site of the long and ring fingers, which the authors believe could be due to the mechanical stresses caused by bowling. He was managed conservatively with observation.

## Introduction

Enthesophytes are bony spurs that form at the insertion sites of ligaments, tendons, or articular capsules on bone, also known as entheses. They can form from mechanical motion and are also associated with inflammatory, degenerative, and metabolic disorders [1-6]. Enthesophytes are often asymptomatic but may be associated with pain and swelling, particularly when caused by inflammatory disorders [3,6-8]. Management commonly includes observation with rest and nonsteroidal anti-inflammatory drugs (NSAIDs). Other treatment options for symptomatic enthesophytes include physical therapy, steroid injections (which remain controversial), disease-modifying antirheumatic drugs (DMARDs) when associated with inflammatory disease, barbotage, and surgical debridement [1,9,10]. In the finger, enthesophytes can develop at the insertion of the flexor digitorum superficialis (FDS). The FDS splits at the level of the proximal phalanx and attaches at the base of the middle phalanx. The function of the FDS is to flex the proximal interphalangeal joint and aid in flexion of the metacarpal phalangeal joint. We present the case of a patient who developed enthesophytes of the long and ring fingers, which we believe may have been associated with bowling. To our knowledge, there are no cases in the literature of finger enthesophytes associated with bowling or any other form of recreational activity that have been reported. 

## Case presentation

Our patient was a 56-year-old right-hand-dominant, otherwise healthy male who worked in material handling in the auto industry and was an avid bowler who bowled with his right hand. He presented to the clinic for evaluation of right ring finger distal interphalangeal (DIP) joint pain, as well as occasional pain in the middle finger while bowling. The pain began and progressed over the prior bowling season and improved once the season ended, though it remained present. 

On examination of the right hand, the patient had large calluses on the radial aspect of the thumb interphalangeal joint and the ulnar aspect of the middle finger DIP joint. He was tender to palpation about the ring finger DIP joint and had firmness to palpation of the flexor tendon over the middle phalanx. He demonstrated full FDS and flexor digitorum profundus function in all fingers with no restriction in range of motion.

**Figure 1:**
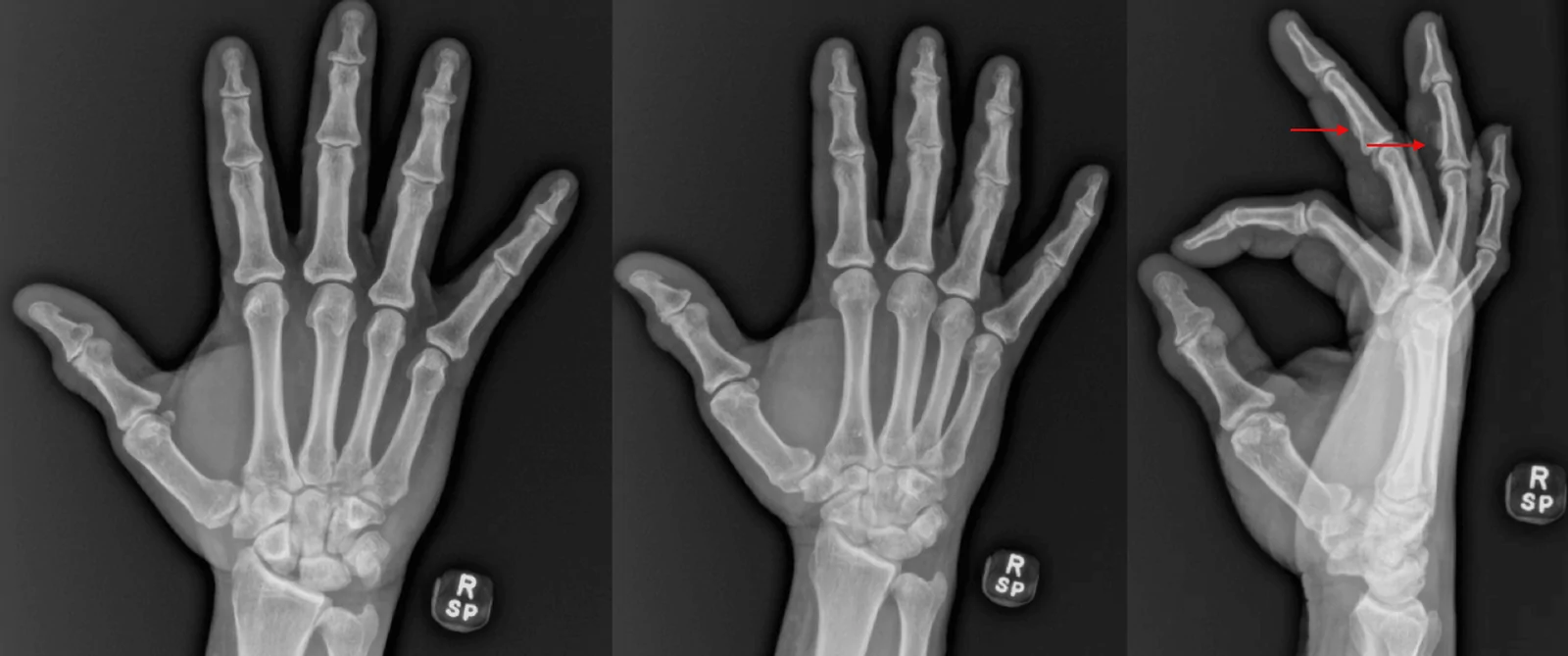
Anteroposterior, oblique, and lateral radiographs of the right hand demonstrate palmar osseous spurs at the base of the middle phalanges of the long and ring fingers (arrows on the lateral view), as well as arthritic changes in the distal interphalangeal joint of the ring finger, including joint space narrowing and a palmar osteophyte. A smaller osseous spur is also present at the base of the middle phalanx of the index finger.

Radiographs of the right hand, seen in Figure 1, demonstrated large osseous lesions extending volarly from the middle phalanx of the long and ring fingers, with the osseous lesion of the ring finger being the most prominent. There was also a smaller osseous lesion on the volar aspect of the base of the middle phalanx of the index finger. These lesions were consistent with enthesophytes at the FDS insertion. Significant osteoarthritis of the ring finger DIP joint was also noted.

Given that the enthesophytes were minimally symptomatic, conservative management was planned. For the ring finger DIP joint osteoarthritis, conservative management with rest and anti-inflammatory gel was also chosen, as he retained a preserved range of motion. The patient was instructed to follow up as needed if his enthesophytes became symptomatic or if his arthritis symptoms failed to improve or worsened.

## Discussion

We hypothesise that a possible aetiology for our patient's development of enthesophytes at the FDS insertion of his long and ring fingers was repetitive mechanical stress. Enthesophytes form at entheses; the sites where ligaments, tendons, and articular cartilage insert into bone. It is hypothesised that enthesophytes develop as a repair mechanism in response to mechanical stress [2-6, 11]. When a tendon or ligament experiences excessive mechanical load, micro-damage occurs at the fibrocartilaginous enthesis. In response to this damage, osteoblasts differentiate and produce new bone, resulting in enthesophyte formation [1, 2, 6]. In bowling, the distal aspects of the middle and ring fingers are inserted into holes in the ball, which may engage the digit as proximally as the proximal interphalangeal joint or as distally as the DIP joint, depending on the bowler’s grip preference. In our patient, repetitive mechanical stress generated by inserting the middle and ring fingers into the ball and releasing it could have driven enthesophyte formation at the FDS insertion. During the acceleration phase of the bowler’s swing and release, great stress is also placed on the proximal interphalangeal joint by firing of the FDS to provide force transmission from the forearm to the distal digit. Common bowling-related injuries described in the literature include De Quervain’s tenosynovitis, lateral and medial epicondylitis, carpal tunnel syndrome, and trigger finger [12].

Enthesophyte formation is thought to begin around age 25 and peak around age 60. It is associated with advancing age and is more common in males than females [4]. In addition to his enthesophytes, our patient also developed osteoarthritis of the ring finger DIP joint. Rogers et al. studied 337 adult skeletons and found a strong positive relationship between enthesophytes and osteophytes, even after accounting for age and sex. They proposed that some individuals may be predisposed “bone formers", though further studies are needed to evaluate this theory [5]. More recent studies have attributed the association between enthesophytes and osteophytes primarily to age [3, 4]. Gibson et al. identified a positive relationship between hand enthesophytes and self-reported diabetes; our patient was not diabetic [3].

In the literature, the majority of reported cases of finger enthesophyte formation have been attributed to inflammatory disorders, particularly seronegative spondyloarthropathies such as psoriatic arthritis [1, 3, 4, 6-8]. In these conditions, widespread inflammation of the enthesis, known as 'enthesitis', can occur. It is hypothesised that the resulting inflammatory cascade, including macrophage and chemokine recruitment, promotes new bone formation [5, 6]. In inflammatory conditions, multiple entheses are typically affected simultaneously, which is generally not the case when mechanical stress is the underlying cause [1]. The standard treatment for enthesitis associated with spondyloarthropathies is DMARDs [1]. Our patient had no known inflammatory disorders.

A common site of enthesophyte formation related to mechanical stress is the calcaneal tuberosity at the Achilles tendon insertion. Calcaneal enthesophytes occur more frequently in the setting of a tight gastrocnemius-soleus complex, which increases tensile loads at the insertion. Given their prevalence, calcaneal enthesophytes have well-described treatment algorithms. The majority are managed nonoperatively with NSAIDs, modified footwear, and stretching exercises. When conservative management fails, surgical options include tendon debridement, calcaneal resection, and osteotomy [1, 9]. In contrast, treatment options for finger enthesophytes arising from mechanical stress are far less well described in the literature. Dimitrakopoulou et al. describe a unique case in which a 23-year-old male sustained an acute avulsion of the left adductor longus origin during a rugby match. Conservative management was initially pursued, but the patient had persistent pain. MRI demonstrated a large 5 cm enthesophyte at the pubic bone at the adductor insertion. He subsequently underwent surgical resection of the enthesophyte, with complete resolution of symptoms [10].

The major limitation of our case report is that we cannot support a causal association between bowling and enthesophyte formation, as this is a single observational case. Other factors that may have contributed to the patient's enthesophyte formation include prior injury such as flexor tendon avulsion, an undiagnosed inflammatory disorder, age, and mechanical stresses from his occupation in material handling in the auto industry. The patient also had a small enthesophyte on his index finger, though it was not as large as those on his ring and long fingers and was asymptomatic. Our patient did not report any prior injuries or history of inflammatory disorders. We did not believe it was necessary to order further inflammatory workup, such as advanced imaging or labs, as the patient was not exhibiting other symptoms. 

## Conclusions

To our knowledge, no cases of finger enthesophytes associated with bowling or other recreational activities have been previously reported. We hypothesize that the enthesophytes of the middle phalanx of the long and ring fingers at the FDS insertion in our patient could have resulted from repetitive mechanical stress caused by bowling. Since this is a single observational case report, our conclusion demonstrates a plausible association rather than establishing a causal relationship. Additional reports and larger observational studies would need to be performed to potentially establish a causal relationship between bowling and enthesophyte formation. This report is intended not as a treatment recommendation for mechanically induced finger enthesophytes, but as a description of a unique radiographic finding.
